# Novel Risk Classification Based on Pyroptosis-Related Genes Defines Immune Microenvironment and Pharmaceutical Landscape for Hepatocellular Carcinoma

**DOI:** 10.3390/cancers14020447

**Published:** 2022-01-17

**Authors:** Jianye Wang, Ying Wang, Marcella Steffani, Christian Stöß, Donna Ankerst, Helmut Friess, Norbert Hüser, Daniel Hartmann

**Affiliations:** 1 Department of Surgery, TUM School of Medicine, Klinikum Rechts Der Isar, Technical University of Munich, 81675 Munich, Germany; jianye.wang@tum.de (J.W.); marcella.steffani@tum.de (M.S.); christian.stoess@tum.de (C.S.); helmut.friess@tum.de (H.F.); norbert.hueser@tum.de (N.H.); 2 Chair of Livestock Biotechnology, School of Life Sciences Weihenstephan, Technical University of Munich, Liesel Beckman Str. 1, 85354 Freising, Germany; medwys123@gmail.com; 3 Departments of Mathematics and Life Science Systems, Technical University of Munich, Boltzmannstr. 3, 85748 Garching, Germany; ankerst@tum.de

**Keywords:** HCC, pyroptosis, prognosis, tumor microenvironment, immunotherapy

## Abstract

**Simple Summary:**

It has been indicated that pyroptosis functions in the development of cancer as well as the orchestration of cancer cell death. Nonetheless, specific roles of pyroptosis-related genes in tumor progression, immune response, prognosis and immunotherapy have, to date, not been thoroughly elucidated. After a comprehensive evaluation of pyroptosis patterns, unsupervised clustering was performed to generate three distinct clusters of 26-gene profiles from HCC. We aimed to establish an efficiency criterion for classifying and predicting patients’ prognosis. After comprehensive analysis of the clustering and risk scoring system, satisfactory sensitivity and specificity are demonstrated, and new insights into the molecular characterization of pyroptosis-related subtypes are contributed.

**Abstract:**

Growing evidence has indicated that pyroptosis functions in the development of cancer. Nonetheless, specific roles of pyroptosis-related genes in tumor progression, immune response, prognosis, and immunotherapy have not been thoroughly elucidated. After a comprehensive evaluation of pyroptosis genes, unsupervised clustering was performed to generate three distinct clusters from hepatocellular carcinoma (HCC) samples. Three distinct pyroptosis-related molecular subtypes comprising three gene clusters that had differential prognostic effects on patient survival were then identified. Immune characteristics analyses revealed diversified immune cell infiltration among the subtypes. Two clusters served as immune-hot phenotypes associated with significantly poorer survival compared to a remaining third immune-cold cluster. Among these, the immune-hot clusters were characterized by abundant adaptive immune cell infiltration, active CD4+ and CD8+ T cells, high total leukocyte counts and tumor growth status, and lower Th17 cell and M2 macrophage densities. Then, risk scores indicated that low-risk patients were more sensitive to anti-tumor therapy. Subsequently, we found a significant correlation between pyroptosis and prognosis in HCC and that pyroptosis genes drive the heterogeneity of the tumor microenvironment. The risk scoring system, based on pyroptosis-related differentially expressed genes, was established to evaluate the individual outcomes and contribute to new insights into the molecular characterization of pyroptosis-related subtypes.

## 1. Introduction

As the sixth most commonly diagnosed cancer and the fourth leading cause of cancer deaths, liver cancer brings intense comorbidity and socio-economic pressure to its patients and support systems worldwide. Hepatocellular carcinoma (HCC) accounts for 85–90% of all primary liver cancers [1,2,3]. In addition to hepatic resection and liver transplantation, systemic therapy, multi-kinase inhibitors, antiangiogenics, and immune checkpoint inhibitors are the foremost effective treatment strategies for HCC [4,5,6]. Many patients are diagnosed at relatively advanced stages of disease and have a poor prognosis. These patients require palliative systemic treatment, which has predominantly consisted of the tyrosine kinase inhibitor over the last decade. Immune check-point inhibitor therapy targeting either the PDL1 or CTLA-4 pathways shows a breakthrough improvement of HCC. HCC is often characterized by intense vascularization by arterial blood vessels that develops secondarily to over-expression of pro-angiogenic vascular endothelial growth factor A and platelet-derived growth factor. Monotherapies might not be satisfactory for patients with advanced HCC. Therefore, a strong rationale for combining ICI and antiangiogenics therapies exists and the approaches have shown efficacy. Of note, despite recent advances in surgery and chemotherapy, the prognosis of HCC is still unsatisfactory, with a recurrence rate of approximately 70% at 5 years after surgery [7]. As more and more mutant genes and epigenetic alterations associated with the occurrence and progression of HCC have been identified, the main focus has been on hepatitis viruses and apoptosis [8,9], including telomerase reverse transcriptase (TERT), tumor protein p53 (TP53), and Catenin Beta 1 (CTNNB1) [10,11]. There remains an urgent need for elucidation of the molecular mechanisms of HCC towards the path of novel effective therapeutic target discovery.

Cell death comprises necroptosis, pyroptosis, ferroptosis, and apoptosis. Pyroptosis is a recently discovered form of caspase-1-triggered programmed cell death (PCD), also known as cellular inflammatory necrosis, characterized by plasma membrane rupture and extracellular release of pro-inflammatory contents (IL-1β, IL-18) [12]. Pyroptosis is a powerful inflammatory mode of lytic cell death caused by various infectious and sterile injuries [13]. Recent studies have indicated that pyroptosis is involved in the occurrence and development of infectious, nervous system-related, and atherosclerotic diseases [14], while also aggravating diabetes, neurodegenerative disease, and liver fibrosis [15,16,17]. Further, pyroptosis plays a role in the pathological process of cancer, thereby impacting tumor development [18]. Ramesh et al. found that human prostate cancers lack caspase-1 gene expression [19]. Caspase-1 deficiency was shown to enhance colorectal tumor formation caused by inflammation in mice [20]. Nevertheless, heterogeneous relationships between pyroptosis and tumors have been reported across different tissues and genetic backgrounds [21]. Pyroptosis was shown to be beneficial to the clinical survival of HCC patients [18]. Inflammatory factors released during the activation of pyroptosis not only enhanced the immunity to pathogenic factors but also inhibited the proliferation and migration of liver cancer cells [22]. However, excessively activated pyroptosis aggravated inflammatory response, resulted in liver damage, and even induced the development of liver fibrosis and liver cancer [16]. In brief, pyroptosis has been shown to act as a double-edged sword, but its correlation to HCC has not been thoroughly elucidated to date. Qualification and quantification of the relationship between pyroptosis and liver disease could provide new strategies for the prevention and treatment of liver disease.

While previous studies have shown that the prognostic signature of HCC is closely related to apoptosis, autophagy, ferroptosis hypoxia, metabolism, and other processes, to date, there is no study focused on specific functions driving the associations [23,24,25,26,27]. Pyroptosis is an intricate multi-step process regulated by a series of pyroptosis-related genes. The overall role of these pyroptosis-related genes in tumor prognosis and immune response remains unknown. Based on pyroptosis-related differentially expressed genes (DEGs), this study aimed to divide clinical samples into three distinct subtypes with different clinical results and tumor microenvironment (TME) immune cell landscapes.

## 2. Materials and Methods

### 2.1. Data Availability and Preprocessing

Clinical follow-up and RNA-seq data from 618 patients with HCC were extracted from the Gene-Expression Omnibus (GEO) database (https://www.ncbi.nlm.nih.gov/geo/query/acc.cgi?acc=gse14520, accessed on 1 December 2019, *n* = 247) and the Cancer Genome Atlas (TCGA, https://portal.gdc.cancer.gov/, accessed on 26 April 2021, *n* = 371) (Table S4 and Table S5). Fragments Per Kilobase Million (FPKM) data from TCGA-Liver Hepatocellular Carcinoma (LIHC) were converted to transcripts per kilobase million (TPM), and normalized by dividing each value by the sum of all FPKM values for each tumor sample, followed by multiplication by 1 × 10^6^. In cases of more than one probe per gene, average values were chosen. Quantile normalization was performed to obtain final expression arrays for analysis. The “ComBat” algorithm from the sva package was used to merge data from the TCGA and GEO databases in order to reduce batch effects. Tumor samples without follow-up information or average gene values = 0 were excluded. Methylation data using Illumina Human Methylation 450 arrays, DNA methylation-based stemness scores (DNAss), and RNA-based stemness scores (RNAss) were downloaded from UCSC Xena (https://xenabrowser.net/datapages/, accessed on 7 August 2019). Another HCC array of 243 samples with expression profiles and clinical information from the Japan International Cancer Genome Consortium (ICGC LIRI-JP) was collected (https://dcc.icgc.org/, accessed on 26 November 2019) and listed in Table S4; in addition, all the overall survival information of all TCGA and GSE14520 datasets were used for survival analysis. Copy number variation (CNV) data were collected from https://gdc.cancer.gov/about-data/publications/panimmune (accessed on 26 April 2021).

### 2.2. Unsupervised Clustering Method of Proptosis-Related Subtypes

An unsupervised clustering method was used as a clustering tool to classify patients. Expression levels from 26 pyroptosis-related genes (AIM2, CASP1, CASP3, CASP4, CASP5, CASP6, CASP8, CASP9, ELANE, GPX4, GSDMB, GSDMD, GSDME, IL-18, IL-1B, IL-6, NLRP1, NLRP2, NLRP3, NOD1, NOD2, PLCG1, PRKACA, PYCARD, SCAF11, TNF) [28,29,30,31,32,33,34,35,36] were extracted from integrated GEO and TCGA datasets for clustering into 2 to 9 clusters using the R ConsensusClusterPlus package. A consistency matrix was created to clarify gene modules and sample clustering numbers, with repeated subsampling under different starting values for 1000 replicates to ensure classification stability [37]. Then, in this study, k values were determined according to the criteria when the cumulative distribution function (CDF) plots reached the approximate stable maximum and the consensus matrix showed the relative clearly diagonal blocks. To better explore the prognostic value of pyroptosis genes, expression profiles of TCGA-LIHC were combined with GSE14520.

### 2.3. Gene Set Variation Analysis (GSVA), Enrichment and Visualization, and Single-Sample Gene Set Enrichment Analysis (ssGSEA)

GSVA was implemented in R to estimate the pyroptosis patterns in biological processes [38] using hallmark gene sets—“h.all.v7.4.symbols”—from the Molecular Signatures Database (MSigDB). Adjusted *p* values of less than 0.05 were filtered as significant. The R pheatmap package was employed to visualize GSVA results, using the ssGSEA to calculate abundances of variate immune cells [38], which were represented by the enrichment scores.

### 2.4. Estimation of Immune Infiltration and Stromal Score

The ESTIMATE algorithm, as a novel method for evaluating the cellularity and CIBERSORT algorithm [39], could yield the ESTIMATE score to predict tumor purity. CIBERSORT is another analytical tool used to estimate a bunch of 22 certain cell types in mixed cell populations [40]. CIBERSORT uses a set of a signature with 547 genes with representative minimal expression values, which act as cell type references. The sum of all the estimated immune cellular scores of each sample was equal to 1.

### 2.5. Generation of DEGs between Pyroptosis Distinct Clusters

A single optimal partition was selected by assessing a consensus matrix and cumulative distribution. The R limma package was applied to identify DEGs between different clusters [41]. The R VennDiagram package was applied to visualize subcluster overlap and determine DEGs stability. DEG-FDR (false discovery rate)-adjusted *p* values of less than 0.05 were then eligible for overlap analysis.

### 2.6. Construction of the Pyroptosis Gene Signature

First, by using the univariate Cox regression model, all the 549 DEGs were analyzed. Furthermore, 300 prognostic-related genes were filtered for subsequent analysis. The patients were then classified to several groups for further analysis by means of the unsupervised clustering method. The consensus clustering algorithm was also used to define the cluster number and stability. Meanwhile, for better evaluation of pyroptosis patterns in tumor samples, the screened DEGs was then extracted to generate a risk scoring system for individual patients, termed as the pyroptosis score. A principal components analysis (PCA) on the 618 samples with the screened DEGs expressions was applied to each cluster with principal components 1 (PC1) and 2 (PC2) regarded as signature scores. Since PCA acts as a dimensional reduction algorithm to downscale modulators with low correlations, the pyroptosis score was defined as the equation: pyroptosis score = Σ (PC1_i_+ PC2_i_), where i represents the expression of each pyroptosis phenotype related gene, similar to the gene expression grade index that is widely used in breast cancer, which focuses on the highly contributed modulators [42].

### 2.7. The Relationship between Immunomodulators (IMs) and Gene Clusters

A total of 78 IMs were enrolled according to a previous study [43], with 3 genes (HLA-DRB3, HLA-DRB4 and KIR2DL2) excluded due to lack of corresponding data. Median normalized expression levels were regarded as representative values for each cluster. Kruskal–Wallis tests were applied to determine significance of subtype median differences. Illumina human methylation 450 dataset of TCGA-LIHC samples was collected from https://portal.gdc.cancer (accessed on 26 April 2021).

### 2.8. The Immunophenoscore (IPS) Analysis

IPS is a scoring scheme based on immune-related gene expression z-scores that are representative of effector cells, immunosuppressive cells, major histocompatibility complex (MHC) molecules, and select immunomodulators. The score ranges from 0 to 10 with higher scores reflecting increased immunogenicity. IPS has been validated for the prediction of patient response to anti-CTLA4 and anti-PD-1 therapies [44]. The IPS data source for HCC patients was downloaded from The Cancer Immunome Atlas (TCIA) (https://tcia.at/home (accessed on 9 May 2021)). The R pRRophetic package, which includes EGFR, multi-kinases, mTOR inhibitors, etc. was applied to estimate drug sensitivity for HCC, defined by the inhibitor concentration where response is reduced by half (IC50) [45]. Then the Wilcoxon signed-rank test was used to perform different analyses.

### 2.9. Statistical Analysis

Spearman and distance correlations were applied to estimate pairwise associations among target parameters, with one-way analysis of variance (ANOVA) and Kruskal–Wallis tests for more than two groups [46]. To investigate alterations of all pyroptosis genes in HCC, the Wilcoxon signed-rank test was performed for the different analyses of gene expression profiles. For survival analysis, the R survminer package was used to determine the optimal cutpoint of the risk score to triage patients into high versus low risk of survival. Similarly, it repeatedly calculated all possible cutpoint and selected the largest rank statistic to divide them into high (or low) tumor mutation burden. The algorithm works by repeatedly testing all potential intersections in silico to find the optimal rank statistics for separating the survival curves of patients below and above the cut-off points of the risk score. The Kaplan–Meier method was utilized to generate survival curves and log-rank tests were used to identify significant differences among subgroups. Patients with detailed clinical data were included for a multivariable prognostic analysis of survival. Specificity and sensitivity of the pyroptosis score were assessed through receiver operating characteristic (ROC) curves, with areas under the curve (AUC) quantified using the R pROC package. ROC analysis was used to validate the feasibility of multiple items (containing risk score prediction model, age, gender, TNM staging). A mutation landscape plot was constructed via the waterfall function of the R maftools package [47]. The R Circos package was employed to plot the CNV landscape in positions of all human chromosomes [48]. All data in this study were analyzed using R software (version 4.0.1).

## 3. Results

### 3.1. Expression and Copy Number Variation (CNV) of Pyroptosis Related Genes in HCC

**Figure 1:**
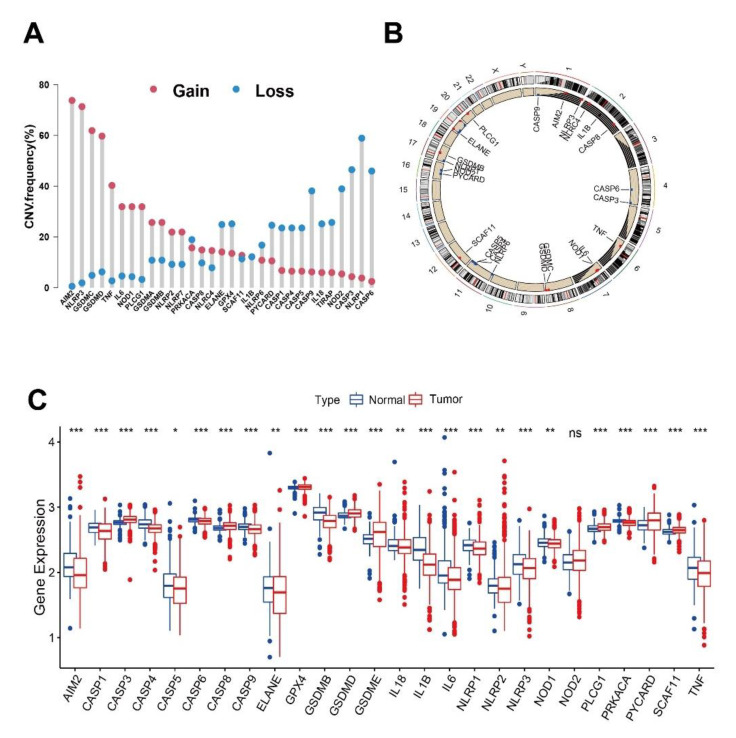
Visualization of the frequency (%) of gains or losses in copy number variation (CNV) from the TCGA database. (**A**) The CNV mutation frequency of the gains (red dot) and losses (blue dot) and (**B**) the location in the chromosome (red represents gain% > loss% and vice versa). (**C**) Gene expression distributions of pyroptosis genes in normal (blue) and tumor (red) samples from TCGA and GSE14520 databases. ns: not significant, * *p* < 0.05, ** *p* < 0.01, *** *p* < 0.001.

A systematic literature search identified genes related to pyroptosis as shown in Table S1, with CNV and the somatic mutation landscape summarized in Figure 1A,B and Figure S1A. With those from GSE14520 being excluded due to a lack of matched CNV and mutation data, only data from TCGA are shown. Among 364 tumor samples with mutation data, 53 (14.6%) experienced mutations of pyroptosis-related genes, where NLRP2 and NLRP3 exhibited the highest mutation frequencies, and AIM2, IL-18, IL-1B, IL-6, NLRP1, NOD1, PYCARD, TIRAP, TNF, CASP1, CASP5, CASP6, CASP9, GPX4, GSDMA, GSDME, PJVK, and SCAF11 had no mutations across all samples (Figure S1A). From CNV analysis of the 26 genes, AIM2, NLRP3, GSDMC, and GSDMD had widespread amplification frequencies, and NLRP1, CASP3, and CASP6 were the most focused on CNV deletion (Figure 1A,B). As shown in Figure 1C, the expression levels of some genes presented the same trends as the CNV alteration (elevated expression in amplification-gain, downregulated expression in deletion-loss in tumor), such as GSDMD, CASP3, PLCG1, and NLRP1, whereas a few genes showed opposite expression trends to the CNV alteration, including AIM2, NLRP3, TNF, and IL-6. In the univariate Cox regression analysis of the optimized gene cutpoints, most showed statistically significant (*p* value < 0.05) associations with survival, except for SCAF11, PYCARD, CASP8, CASP6, CASP3, AIM2, IL-18, NOD1, and GSDMD (Figure S1B) and the magnitudes of effect (hazard ratio) of all the genes are summarized in Figure S1C (integrated dataset) and Figure S1E,G (independent datasets). Some genes with high expression levels were associated with better outcomes (e.g., NLRP1, CASP9) and others presented worse prognosis (e.g., PLCG1, GSDME). Interestingly, for separated data analysis, as the Figure S1D–G (left part D and E: TCGA-LIHC; right part F and G: GSE14520) show, most pyroptosis genes had the same statistically significant trend as the integrated analysis. Except for some minor variations, e.g., CASP5, GSDME, NLRP1, NOD2, and PYCARD showed no differences in the tumor samples of TCGA-LIHC (Figure S1D), and ELANE, NLRP1, and NOD2 showed differences in the tumor samples of GSE14520 (Figure S1F), whereas only NOD2 showed no differences in the tumor samples of the integrated data. Therefore, the alterations of expression and genetic variation in pyroptosis genes play a crucial role in HCC.

### 3.2. Pyroptosis Patterns Mediated by 26 Regulators

**Figure 2:**
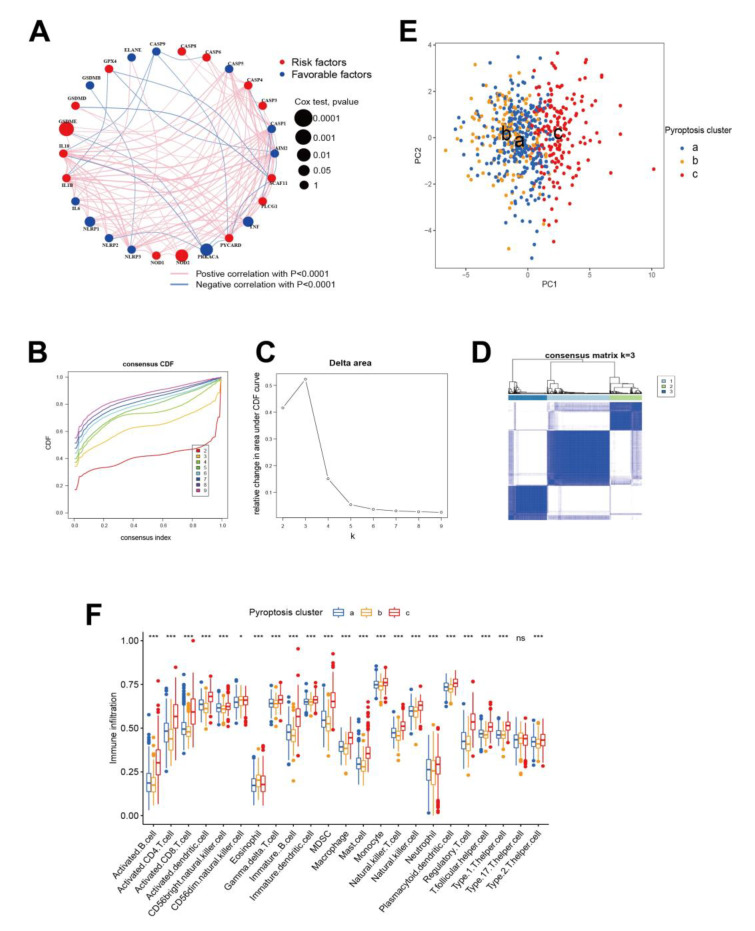
Identification of potential immune subtypes of HCC based on pyroptosis genes. (**A**) The correlation network of the pyroptosis-related genes (red line: positive correlation; blue line: negative correlation. The size (*p* value from 0.001 to 1) of the dot reflects the strength of the relationship between each gene and prognosis). The colors of dots represent protective (blue) and risk roles (red), (**B**) cumulative distribution function (CDF) curve, (**C**) cumulative delta area under CDF for optimum decision of k value, and (**D**) sample clustering heat map (k = 3). (**E**) PCA plots of clusters A (blue), B (yellow), and A (red). (**F**) Immune landscape of immune infiltration of three pattern clusters from ssGSEA. ns: not significant, * *p* < 0.05, *** *p* < 0.001.

Based on the univariate Cox regression analysis of pyroptosis modulators and clinical outcomes, Figure 2A provides a comprehensive landscape correlation network showing that CASP9, GPX4, GSDMB, and PRKACA only shared a common opposite trend expression to other genes, while CASP5, CASP3, TNF, NOD2, NOD1, NLRP1, and IL-6 expression were positively related to others. Tumors with high expressions of writer genes NLRP2, NLRP3, PYCARD, PLCG1 GSDME, IL-18, GSDMD, and SCAF11 exhibited dual correlations to other genes. Likewise, separated correlation network analysis exhibited partial different gene connection (TCGA-LIHC: Figure S2E; GSE14520: Figure S2H). The results indicated that pyroptosis genes were implicated in cancer pathogenesis. Based on this hypothesis, we utilized consensus clustering analysis to stratify samples with the expression of pyroptosis genes. The consensus distributions for k (2 to 9) are showing in cumulative distribution function (CDF) plots (Figure 2B). The consensus matrix indicated that the unsupervised algorithm, based on the pyroptosis regulators, could clearly distinguish the samples; this was integrated with the consensus matrix of k (2 to 9) (Figure S2A), and the area under the CDF (Figure 2C). The optimal number of gene pyroptosis clusters was three. The three clusters showed distinct modification patterns (Figure 2D), including 290, 149, and 179 tumor samples, respectively. The three clusters had no significant association with survival (*p* value > 0.05, Figure S2C, same in individual TCGA-LIHC: Figure S2G, and GSE14520: Figure S2J). Figure S2D shows the survival prediction values of the common clinical characteristics age, gender, and cancer stage after applying the univariate Cox proportional hazards model, only the TNM stage has significant value for outcome prediction in this study (all stage *p* < 0.05). Unsupervised clustering discovered for the expression of pyroptosis patterns suggested that three clusters were primarily clustered by PCA (Figure 2E; similarly in individual TCGA-LIHC (Figure S2F) and GSE14520 (Figure S2I)). Pyroptosis has been reported to play important role in the tumor immune microenvironment. Therefore, we investigated the relationship between pyroptosis clusters and tumor immune cells. The ssGSEA algorithm, based on pyroptosis patterns, indicated that almost all immune infiltration cells were separated by pyroptosis patterns (Figure 2F). Whereas GSDME, PLCG1, and CASP8 showed a higher expression in pyroptosis cluster A, NLRP1, IL-6, NOD1, PYCARD, CASP1, CASP4, CASP5, TNF, AIM2, IL-18, IL-1B, NLRP3, NLRP2, and NOD2 tended to exhibit higher levels in pyroptosis cluster C (Figure S2B).

### 3.3. Characterization of Pyroptosis Gene Subtypes

**Figure 3:**
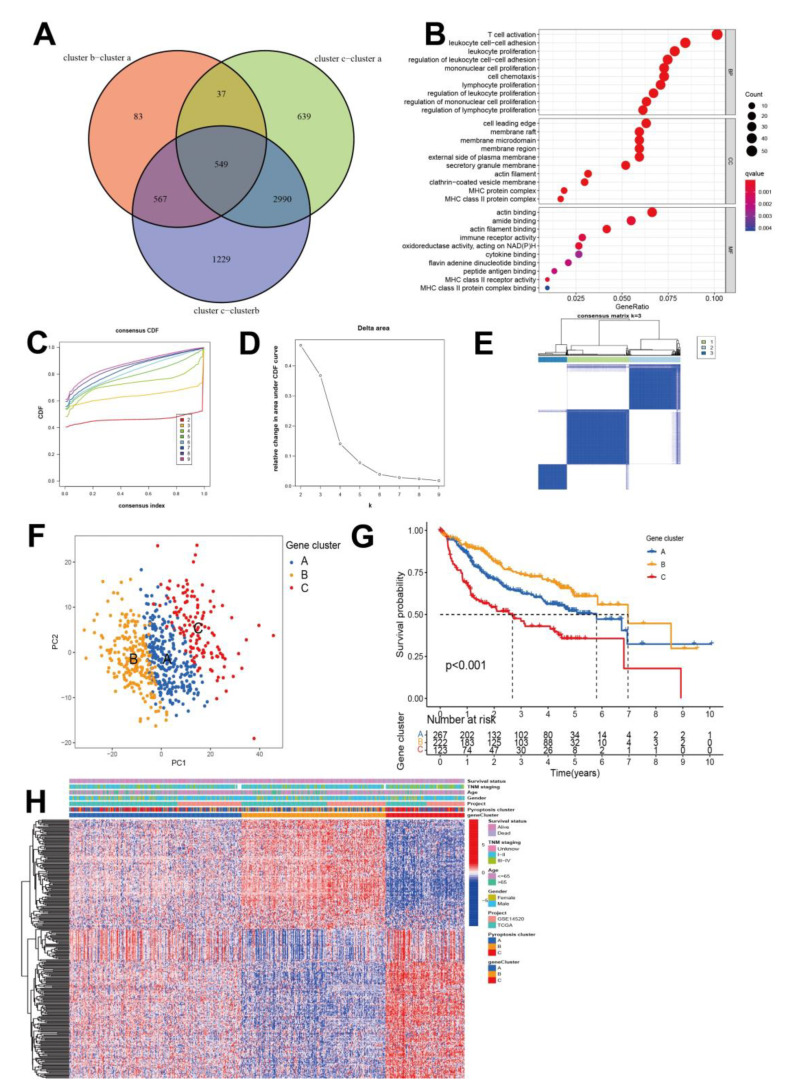
Identification of subtypes based on differentially expressed genes (DEGs). (**A**) Overlapping analyses of significant genes using a Venn diagram. (**B**) GO analysis of overlapping DEGs, (the distance from left *y* axis means gene ratio, the size means the enriched gene counts). Construction of gene clusters, an unsupervised clustering of DEGs in two cohorts’ projects to classify different genomic subtypes, termed gene clusters A–C, respectively. (**C**) Cumulative distribution function curve, (**D**) cumulative delta area under CDF of k = 1 to 9, and (**E**) Sample clustering heat map (k = 3). (**F**) PCA plot of gene clusters A (blue), B (yellow), and C (red). (**G**) Survival analysis of gene clusters A (blue), B (yellow), and C (red). (**H**) The pyroptosis clusters, gene clusters, age, gender, tumor stage, survival status, and projects were used as patient annotations.

**Figure 4:**
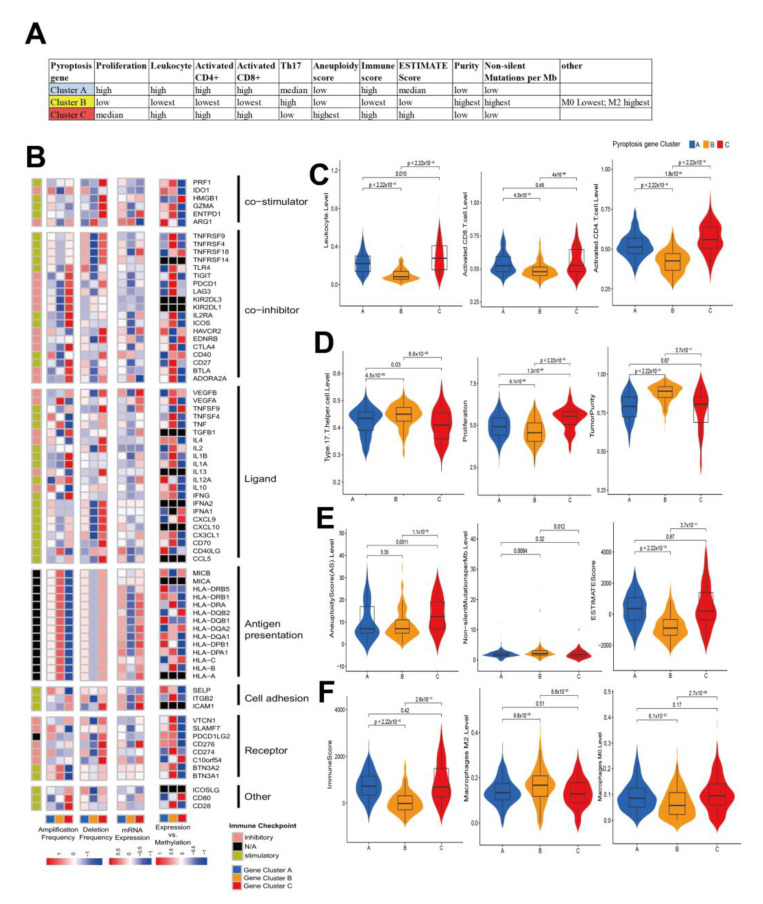
Immune features of three distinct clusters. (**A**) Key characteristics of pyroptosis clusters, (**B**) Regulation of immunomodulators (IMs) of three clusters, from left to right: amplification frequency and deletion frequency, mRNA expression (the median normalized value of each gene was selected as the representative expression of each gene cluster), and their methylation (the correlation between gene expression and DNA-methylation beta-value). The violin plot depicts the differences in key factors between the gene clusters. (**C**) Leukocytes, activated CD8+ T cells, and activated CD4+ T cells; (**D**) Th17 helper cells, proliferation, and tumor purity; (**E**) aneuploidy score, non-silent mutations per Mb, and ESTIMATE score; (**F**) immune score, M2 macrophages, and M0 macrophages.

Table S2 shows the identified DEGs among the groups (Table S2). Subsequently, 549 pyroptosis pattern-related DEGs were recognized (Figure 3A). GO enrichment analysis revealed that these signature genes were related to the biological processes of membrane-related reactions and immune regulation (Figure 3B). This evidence indicated that these DEGs might contribute to the heterogeneity and immune-omics characteristics of HCC. We aimed to use random forest algorithms to reduce the redundant genes to extract the phenotype signatures (*p* < 0.05). A univariate Cox regression analysis filtered 300 prognosis-related genes. For mining the regulate mechanism, they were subsequently subjected to unsupervised clustering analysis in order to divide HCC patients into different genomic subgroups based on the obtained 300 genes (Figure S3A), where three clusters were again selected as the optimal number and each sample in a cluster possessed a high correlation (Figure 3C–E). Clusters A (*n* = 270), B (*n* = 223) and C (*n* = 125) were generated, with the PCA plot verifying that they subdivided patients into three prognostic groups (*p* < 0.001, Figure 3F). Consistent with Figure 2A, verification analysis of the pyroptosis genes (Figure S4A) in the three gene clusters revealed that CASP9, GPX4, GSDMB, and PRKACA had the highest expression levels in cluster B. In addition, analyses of overall survival showed that patients in cluster B had more favorable survival, with significantly longer median survival times compared to the other two clusters (Figure 3G). Patients with higher rates of death and advanced stage III and IV were characterized by gene cluster C patterns (Figure 3H). For immune infiltration analysis, the ESTIMATE and CIBERSORT algorithms were used to recognize immune cell infiltration and related scoring in each subgroup of patients (Figure S4B). Noteworthy, after the same analyses’ procedures, the same three clusters were generated and identified as survival predictors in separate datasets (Figure S3D,G). Notably, as concluded in Figure 4A,C–F, patients in cluster B presented a lower proliferation rate, leukocyte score, activated CD4+ cell, activated CD8+ T cell, ImmuneScore, ESTIMATEScore, and M0 macrophage level, but had the highest Th17 cell and M2 macrophage levels, whereas patients in both clusters A and C had higher numbers in these indices, and higher tumor purity and non-silent mutations per Mb. This indicated that the immune-cold but enriched polarized M2 macrophage cluster B showed improved clinical outcomes compared to the immune-warm clusters A and C. IMs were reported to be critical checkpoints for the application of tumor immune therapy in the clinic [49]. Pairwise Kruskal–Wallis tests showed that CNVs affected most IMs and varied consistently according to the pyroptosis gene types, where cluster C showed high frequencies of CNVs and cluster B showed lower rates (Figure 4B). Expression profiles, CNVs, and correlations between respective DNA methylation and mRNA expression levels of IMs largely segregated tumors according to the three clusters (Figure 4B). Additionally, immune checkpoints CTLA4, TNFRSF9, TNFRSF4, TNFRSF18, TNFRSF14, and TIGIT were significantly lower expressed in cluster B compared to the other clusters (Figure S4C). For CNVs, clusters A and C exhibited frequent amplification and deletion of most IMs. Cluster B had lower IM expression levels, which were positively correlated with DNA methylation levels. GSVA was applied to explore the activity of biological processes among the three clusters (Figure S4D and Table S3). Cluster C was distinctly enriched in pathways associated with immune responses, including the interferon gamma response, the inflammatory response, and the complementary response. Meanwhile, cluster B was markedly related to immunosuppression processes.

### 3.4. Generation of Pyroptosis Gene Signatures and Correlations with Clinical Parameters

**Figure 5:**
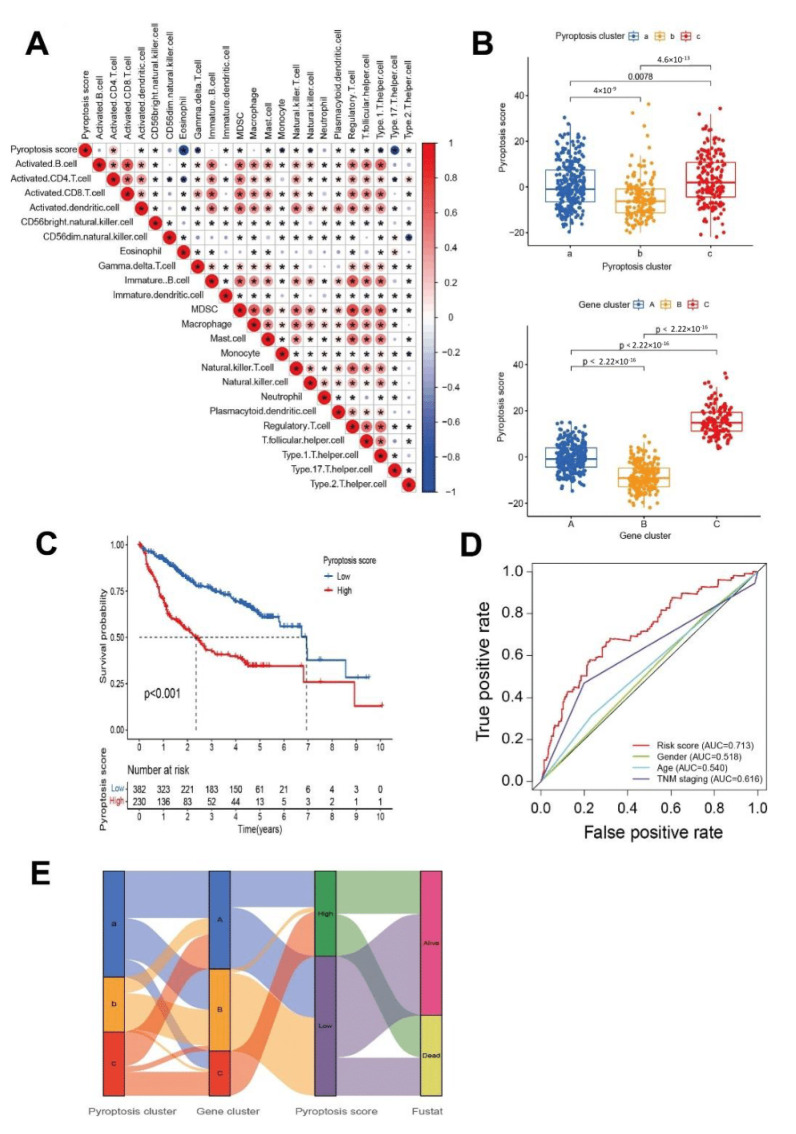
Construction of pyroptosis risk signature. (**A**) Correlation analysis of immune cells and pyroptosis risk score (Red represents positive correlation, blue represents negative correlation; * represents *p* < 0.05). (**B**) Pyroptosis score distributions of all HCC patients in pyroptosis clusters a–c and gene clusters A–C. The Kruskal–Wallis test was used to compare the difference between the three clusters (*p* < 0.001). (**C**) Kaplan–Meier overall survival (OS) analysis of high (red) and low (blue) pyroptosis risk scores. (**D**) ROC curves for evaluation of clinical characteristics (from top to bottom are risk score, TNM staging, age, and gender). (**E**) The Sankey diagram shows the flow diagram of our investigation. The width of the bands is proportional to the flow rate in each part, with a survival curve for risk score classification of HCC patients.

**Figure 6:**
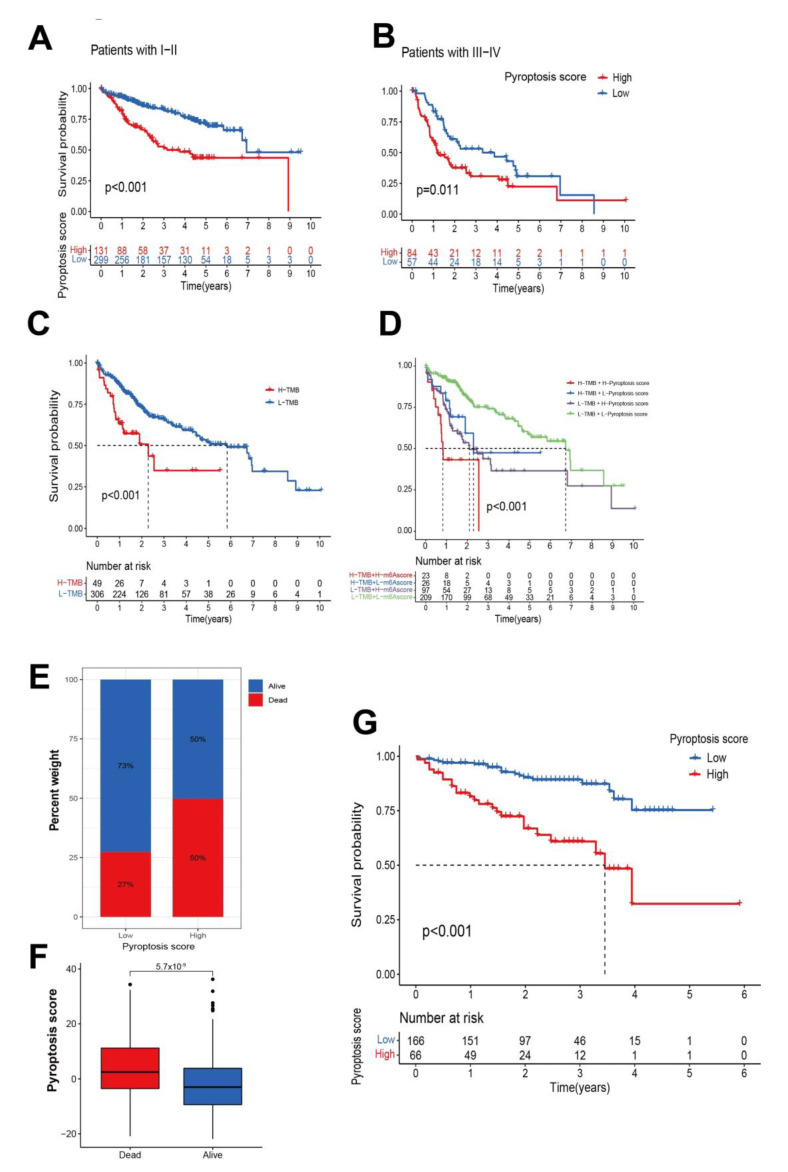
Clinical verification of pyroptosis scores. Overall survival analysis of patients with (**A**) TMN stage I–II and (**B**) TMN stage III–IV categorized in the high- and low- risk groups, respectively, (**C**) Overall survival analysis of high-TMB (red) and low-TMB (blue) tumors, and (**D**) tumors subdivided by the pyroptosis risk score. (**E**,**F**) Distribution and visualization of the follow-up of all patients in the two pyroptosis risk groups. (**G**) Further verification of the risk classification of HCC patients using external ICGC data sets.

To verify whether the classification method was valid for the pyroptosis risk score, the correlation analysis was repeated for each immune cell infiltration in individual patients by using the same pyroptosis risk score (Figure 5A). Kruskal–Wallis tests showed significant differences in pyroptosis scores among clusters (Figure 5B). Prognostic values of the pyroptosis score in patients with HCC were explored by dividing patients into high or low pyroptosis score groups, with the optimal cut-off value determined as 1.58. Patients with high pyroptosis scores above 1.58 demonstrated significant survival impairment (*p* value < 0.001, Figure 5C). ROC curves demonstrated discrimination of the risk score criterion for separating patients with good versus poor survival, producing improved AUCs to TNM staging and other clinical characteristics (Figure 5D). Pyroptosis pattern clusters, pyroptosis gene clusters, risk score groups, and future states are summarized in a Sankey diagram (Figure 5E), a methodology to evaluate the pyroptosis genes accurately for individual patients. Changes in attributes of individual HCC patients are visualized with an alluvial diagram, showing that most of gene cluster B was linked to a low-risk score and higher survival. Correlations between pyroptosis scores and known biological processes were analyzed to better demonstrate features of the pyroptosis gene signature. Overall survival analysis of patients with TMN stage I–II and with TMN stage III–IV, subdivided into high- and low-risk groups, was performed, showing significant separation with *p*-values < 0.001 and 0.011, respectively (Figure 6A,B). Overall survival analysis of high-tumor mutation burden (TMB, red) and low-TMB (blue) (cutoff value = 2.947) (Figure 6C) subdivided according to the pyroptosis risk score (Figure 6D) suggested the independence of the score’s predictive value to TMB. Visual distribution and visualization of the follow-up event of all the patients in the two pyroptosis risk groups shows the significant distribution of patients’ outcomes in two different risk groups (Figure 6E,F). To further evaluate the prognostic value of the risk scoring system of the pyroptosis-related genes, its performance was validated in external ICGC data sets (Figure 6G). Nevertheless, though independent risk scoring systems both exhibited clinical values (Figure S6A,D), in terms of the external verification analysis, the risk-scoring classification generated from TCGA-LIHC did not divide GSE14520 patients (Figure S6B) and ICGC (Figure S4C) into two statistically different groups. Meanwhile, though the risk-scoring classification generated from GSE14520 did divide TCGA-LIHC patients into groups with distinctly different prognoses (Figure S6E), it also failed in predicting ICGC patients (Figure S6F).

### 3.5. Genomic Characteristics of Pyroptosis Signatures

**Figure 7:**
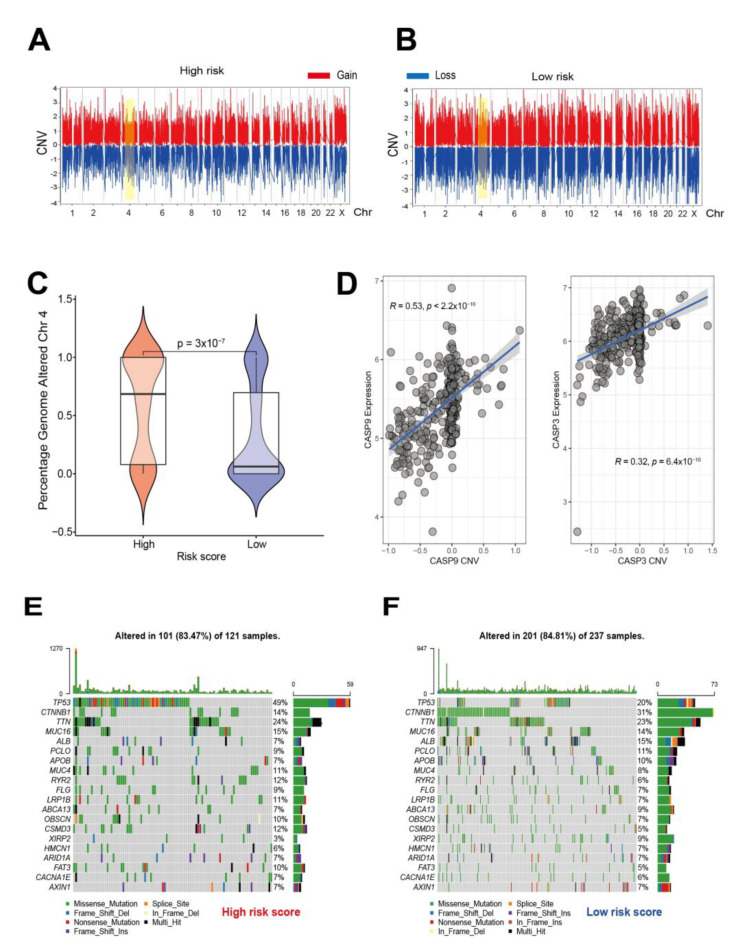
Comparison of the genetic characteristics of the two risk groups based on TCGA cohort. High (**A**) and low (**B**) pyroptosis score groups exhibited different CNV features across the whole chromosome. (**C**) The percentage frequency of genome alteration on chromosome 4 between the two score groups. (**D**) Correlation between CNV and gene expression in CASP9 and CASP3. Somatic mutation waterfall plot in (**E**) the high-risk, and (**F**) low-risk score groups. Each column represents an individual sample. The upper bar plot indicates TMB and the number on the right shows the mutation frequency. The right bar plot shows the proportion of each variant type.

To better compare the CNV of genomic segments in the chromosomes of two high- and low-risk groups, the distributions of segment scores across all chromosomes in Figure 7A,B and Figure S7 show significant differences on chromosome 4. Since these two risk groups have distinct immune infiltration features, pyroptosis genes on chromosome 4 were further analyzed, showing that the immune-related CASP9 and CASP3 were significantly up-regulated in the high score group (Figure 7C), and positively associated with their CNV (Figure 7D). Differences in distributions of somatic mutations between high and low pyroptosis risk scores in the TCGA-LIHC cohort were explored using the maftools package (Figure 7E,F). Although there were similar degrees of somatic mutation landscape in the high- (83.47%) and low-risk (84.81%) score groups in the TCGA cohort, there was a higher TP53 possibility rate (49%) in the high-risk score group as compared to the low-risk score group (20%). TP53 and CTNNB showed the largest alteration between high-and low-risk patients, but no significant differences were observed in terms of TTN and MUC16.

### 3.6. Predictive Response of Pyroptosis Risk Score to Immunotherapy and Systemic Therapy

**Figure 8:**
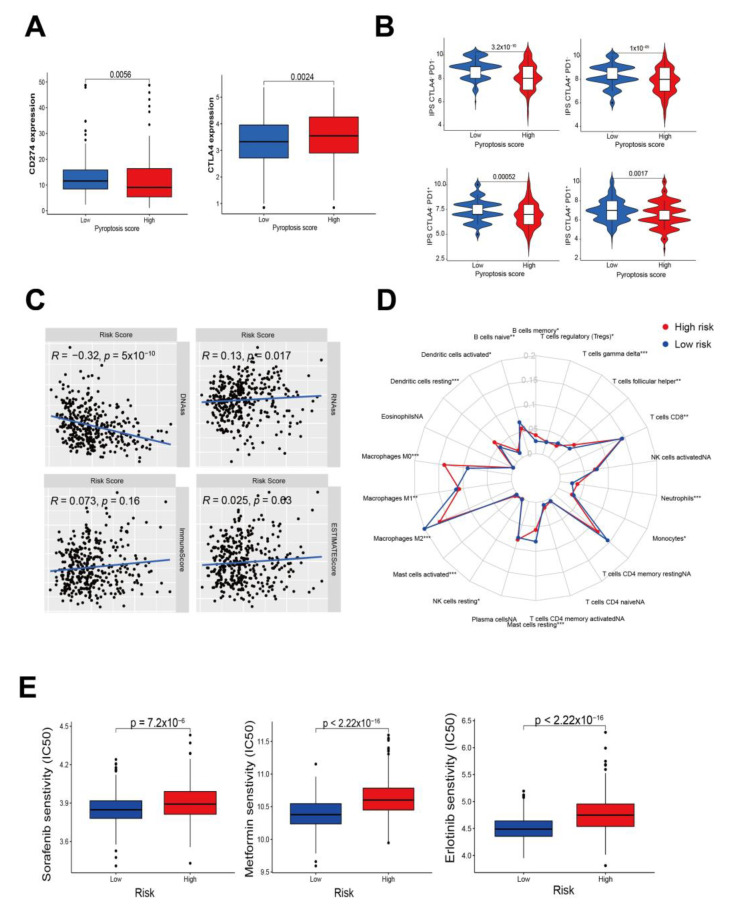
Pyroptosis risk scores in the prediction of immunotherapy. (**A**) Immunological checkpoint CD274 and CTLA4 expression in low- and high-risk groups. (**B**) Immunological checkpoint therapy prediction of risk score in four-CLTA4 and PD1 subtype groups. (**C**) Correlation plot between pyroptosis risk score and DNA stemness score (DNAss), RNA stemness score (RNAss), stromal score, immune score, and ESTMATE score. (**D**) * p < 0.05, ** p < 0.01, *** p < 0.001. Radar plot showing cell infiltration from the CIBERSORT procedure. Each section of the graph represents one of the 22 human cell phenotypes, with each point representing the mean proportion of each cell population in the high-risk (red line) and low-risk groups (blue line). (**E**) Sensitivity analysis of three pharmaceutical therapy in patients at high and low risk.

Finally, as immunotherapy is emerging as a critical breakthrough for tumor treatment, immune checkpoint genes, such as PDL1 and CTLA4, were compared among the two different risk groups. In the low-risk group, the PDL1 levels were higher, while the CTLA4 levels were lower (Figure 8A). Meanwhile, neither positive nor negative levels of CTLA4 in tumor samples were present, and IPS showed a higher level in the low-risk group (Figure 8B). Interestingly, among these scores, including gene expression-based stemness scores (mRNAss), DNA methylation-based stemness scores (mDNAss), stromal scores, immune scores, and ESTIMATE scores, only the DNAss were negatively correlated to risk scores (Figure 8C). Accordingly, the risk score was positively associated with M0 macrophages, but polarized M2 macrophages were more strongly associated with lower risk scores (Figure 8D and Figure S5A–P). Moreover, patients with low risk scores showed more sensitivity (IC50) to sorafenib, metformin, and erlotinib (all *p* < 0.05) (Figure 8E). The above results indicate that the risk score might be of benefit in terms of efficiently predicting the current systemic treatment for HCC.

## 4. Discussion

There is an urgent need to identify novel molecular targets to improve the diagnosis and prognosis of HCC. In recent years, cumulative studies have demonstrated that pyroptosis plays an essential role in inflammation and antitumor immune regulation. To date, most studies have focused on a single pyroptosis-related gene or a single modulator; however, pyroptosis is a complicated multi-step process regulated by a series of genes. The tumor characteristics mediated by the integration of multiple pyroptosis modulators are not yet fully understood, and investigating pyroptosis-related genes may help to clarify the mechanism behind the high heterogeneity of HCC. Therefore, in this study, we systematically profiled pyroptosis-related DEGs and established a novel risk classification in order to define the immune landscape in HCC.

Due to the difference in the data depth and samples between the two datasets, there are various clusters generated, which subsequentially leads to the obtaining of different DEGs and, consequently, two different scoring systems. However, risk scoring systems generated from separated analyses fail in predicting external cohorts. This study aimed to integrate the transcriptional profiling of 26 pyroptosis-related genes that have been proposed to be potential HCC-related genes. The mRNA expression levels showed that most of these genes were differentially expressed between normal and tumor tissue. Pyroptosis, as a proinflammatory form of PCD, plays a dual role in tumor progression. On the one hand, the proliferation and migration of tumor cells can be inhibited by inducing cell inflammatory death. On the other hand, aggravated pro-inflammatory death caused by excessive activation of pyroptosis forms a TME that is suitable for tumor growth, thereby promoting tumor growth [50]. Hence, we established pyroptosis-related subtypes based on unsupervised clustering of the pooled TCGA and GEO databases. Even though there were no differences in clinical prognosis, there were significant differences in immune infiltration among the three pyroptosis clusters, indicating that pyroptosis can regulate the composition of the tumor immune microenvironment.

Long-term exposure of tissues and/or cells to an inflammatory environment increases the risk of cancer [51]. Generally, most forms of PCD maintain the integrity of the cell plasma membrane without releasing its contents, generating no direct inflammatory response; however, pyroptosis is characterized by the opposite response, with the release of intense inflammatory mediators IL-1 and IL-18, which leads to inflammatory “necrosis”. Patients belonging to pyroptosis gene cluster B showed prolonged survival but lower pyroptosis gene expression levels when compared to clusters A and C. This is consistent with our results showing the positive correlation between the reduction in pyroptosis-related inflammatory death and clinical prognosis.

The liver is the largest immune-related organ, and the immune system plays a decisive role in tumorigenesis [52]. There are three immune phenotypes of the HCC tumor environment: immune rejection, immune inflammation, and immune desert [53]. To further explore the potential prognostic significance of the combination of the pyroptosis score system and immune status in HCC, we identified three pyroptosis modification patterns with significantly different immune landscapes, characterized by differences in CD4+ T cells, CD8+ T cells, Th17 characteristics, overall cell proliferation, aneuploidy, changes in numbers of non-silent mutations per Mb, and immunoregulatory gene expression. Gene cluster B presented an immune-cold phenotype with the lowest proliferation, suggesting low tumor growth. Moreover, we observed that gene cluster B shows a high density of Th17 cells and M2 cells, which is in line with a previous study showing that Th17 expression is generally associated with improved tumor prognosis [54]. Th17 cells were reported to inhibit growth and metastasis via the stimulation of cytotoxic T cells and natural killer cells within the TME [55]. In the HCC microenvironment, Th17 cells have a non-pathogenic phenotype and produce anti-inflammatory cytokines [56,57,58]. Classical M1 macrophages engage the inflammatory response and anti-tumor immunity, while alternative M2 macrophages are defined by their anti-inflammatory properties in the immunosuppressive microenvironment [58]. Previous studies have shown that, due to the role of tumor-associated macrophages in immune invasion, higher levels of tumor-activated macrophages are positively associated with poor prognosis in HCC patients [49,59]. Conversely, gene clusters A and C showed an immune-inflammation phenotype with high levels of leukocyte, CD4+ T cell, and CD8+ T cell infiltration, and exhibited high proliferation, suggesting high tumor growth in patients in these clusters. Chronic inflammation in the TME plays a vital role in the development of tumors, and pro-inflammatory cell death can generate a microenvironment that is suitable for tumor growth [60]. Consequently, it is not surprising that gene clusters A and C exhibit activated immunity but had low survival rates. Based on the results of our analyses, it is reasonable to speculate that pyroptosis mediates clinical results by regulating the composition of the TME. In line with these findings, our results revealed that the active inflammatory response in the gene clusters A and C (immune-hot phenotype) was correlated with poor prognosis and could trigger tumor cell immune evasion under a particular TME, imparting resistance to immunotherapy. The inhibition of the pyroptosis-related inflammatory response in samples from gene cluster B (immune-cold phenotype) is responsible for the favorable prognosis.

Another pyroptosis gene scoring system was then constructed in order to predict HCC patients’ survival and its relationship with the TME. Patients with a high-risk score showed a higher frequency of CNV alterations on chromosome 4, as suggested by a study showing that losses of 4q were the most common changes found in medullary thyroid carcinoma [61]. Meanwhile, our study also revealed that immunotherapy is more efficient in low-risk patients. To further evaluate the prognostic value of the regulatory factors of these pyroptosis-related genes, we generated a scoring system that fully classified the HCC patients into high- and low-risk groups according to pyroptosis-related DEGs. Additionally, we validated the stability and reliability of the scoring system using the ICGC cohort. Notably, our current study was indirectly evaluated by the public expression profile database of each patient, despite the exploring and validating data. However, further complete external validation of the identified clusters is still needed.

Cancer immunotherapies based on the targeting of immune checkpoints (PD-1, PDL1, and CTLA4) have shown clinical value in various cancer types [62]. Increased levels of immune checkpoint proteins suppress the anti-tumor immune response of T cells, and the development of drugs that inhibit these proteins has led to significant progress in the treatment of HCC. In this study, we found that high expression of CTLA4, TNFR, and TIGIT in the immune-cold phenotype was associated with an improved clinical prognosis. The higher expression of these immune checkpoints may limit the activity of cytotoxic immune cells in the TME, causing these cells to enter a state of exhaustion [63]. This not only indicates that the pyroptosis score has the potential to predict the efficacy of CTLA4 immunotherapy, but more importantly, it emphasizes the importance of pyroptosis in shaping tumor immunity. Immunotherapy is gradually becoming a revolutionary and promising cancer treatment method. Antitumor drugs such as sorafenib and metformin are recommended for HCC treatment [64]. Thus, we evaluated the sensitivity of HCC to these drugs, showing that low-risk tumors were more sensitive to the drugs, which indicates that the patients in the low-risk group may benefit from these chemotherapeutic drugs as well as immunotherapy. These results may also partially explain why low-risk patients may have a better overall prognosis.

## 5. Conclusions

This study developed a novel risk classification system based on pyroptosis-related DEGs that serves as an independent and robust prognostic biomarker for predicting patient outcomes. Our work classified the patients into different clusters based on the pyroptosis-related genes, which made it possible to reveal an underlying mechanism that has been previously studied in this field but is not currently applied clinically, and thus, the results obtained in this study could provide a clue for further research on the relationship between pyroptosis and HCC. The constructed signature has the potential to enhance the understanding of pyroptosis-related immune microenvironments and identify immunotherapy strategies for HCC.

## Data Availability

Not applicable.

## Supplementary Materials

The following are available online at https://www.mdpi.com/article/10.3390/cancers14020447/s1, Figure S1: General overview of pyroptosis-related genes, Figure S2: Characteristics of pyroptosis patterns, Figure S3: Identification of DEG-related subtypes, Figure S4: The expression distribution and GSVA enrichment features of gene clusters A–C, Figure S5: The association between pyroptosis risk scores and different immune subtypes, Figure S6: Cross verification of high (red) and low (blue) pyroptosis risk scoring system in overall survival analyses among separated databases, Figure S7: Statistical analysis of CNVs in whole chromosomes; Table S1: Basic information of pyroptosis genes in two hepatocellular carcinoma datasets, Table S2: Differentially expressed genes among three pyroptosis-clusters, Table S3: GSVA enrichment features of the hallmark gene set among the three gene clusters, Table S4: Identity document of all patients in this study, Table S5: Clinical characteristics of the patients employed in this study.
