# Supplementary material for: Novel Risk Classification Based on Pyroptosis-Related Genes Defines Immune Microenvironment and Pharmaceutical Landscape for Hepatocellular Carcinoma
Source: Cancers (Basel). 2022 Jan 17;14(2):447. doi: 10.3390/cancers14020447 (PMC8773536; doi:10.3390/cancers14020447)
Supplement: Supplementary file 1 [file cancers-14-00447-s001.zip › Supplemental Figures.pdf]

Supplemental Material:

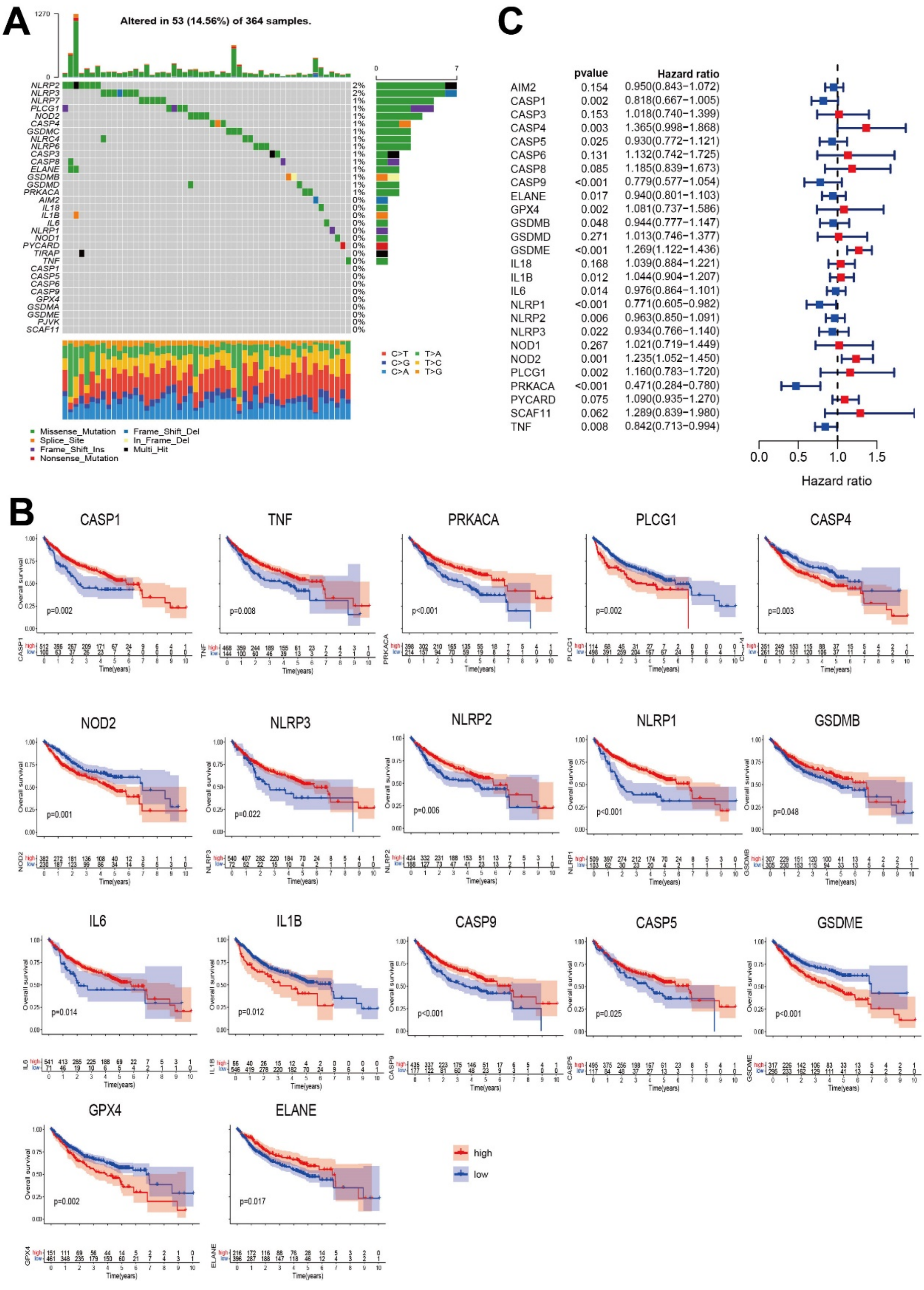

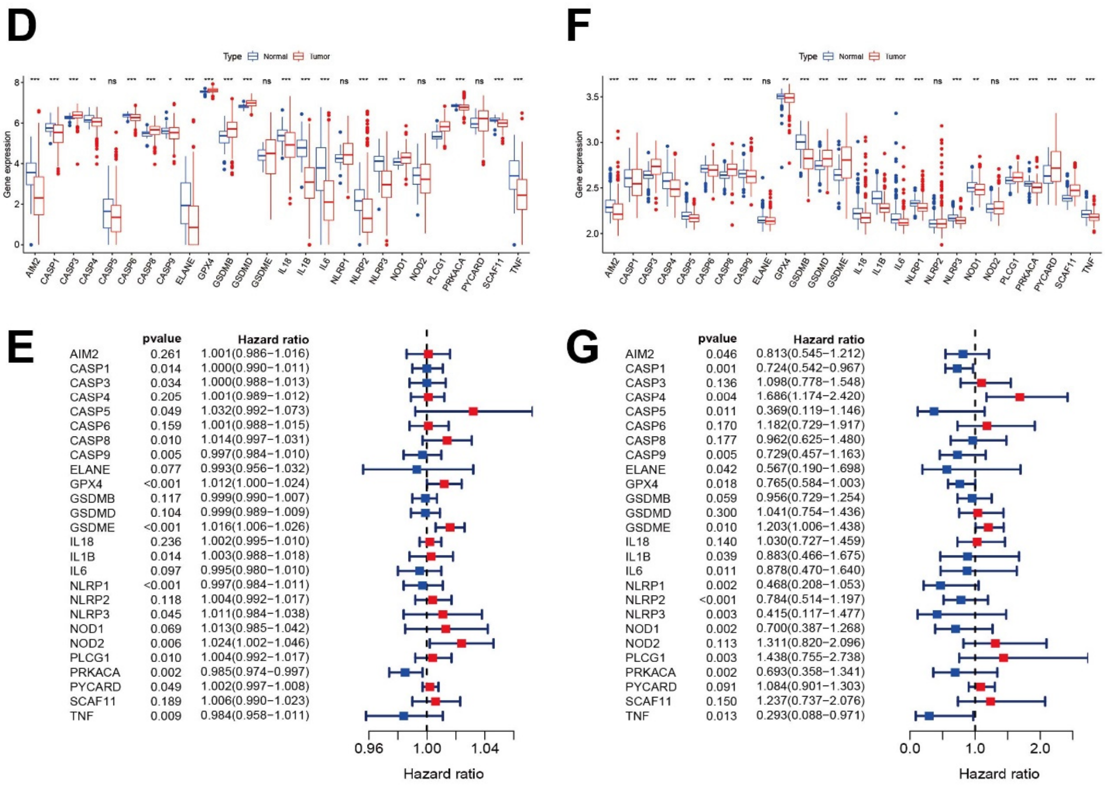

Figure S1. General overview of pyroptosis-related genes. (**A**) Somatic mutation features of each of the pyroptosis genes in TCGA cohort. (**B**) Kaplan-Meier survival analysis of patients with high- (red) and low- (blue) expression for each pyroptosis-related gene in all patients with clinical information available. CASP1, TNF, PRKACA, PLCG1, CASP4, NOD2, NLRP3, NLRP2, NLRP1, GSDMB, IL-6, IL-1B, CASP9, CASP5, GSDME,.GPX4, ELANE (all $p < 0.05$). (**C**) Forest plots based on the results of Kaplan-Meier analysis between gene expression level and overall survival in integrated dataset; Separated analyses of pyroptosis genes, (**D**,**F**) Gene expression distributions of pyroptosis genes in normal (blue) and tumor (red) samples. (**E,G**) Forest plots based on the results of Kaplan-Meier analysis between gene expression level and overall survival in separated dataset; (**D**,**E**) TCGA-LIHC; (**F,G**) GSE14520.

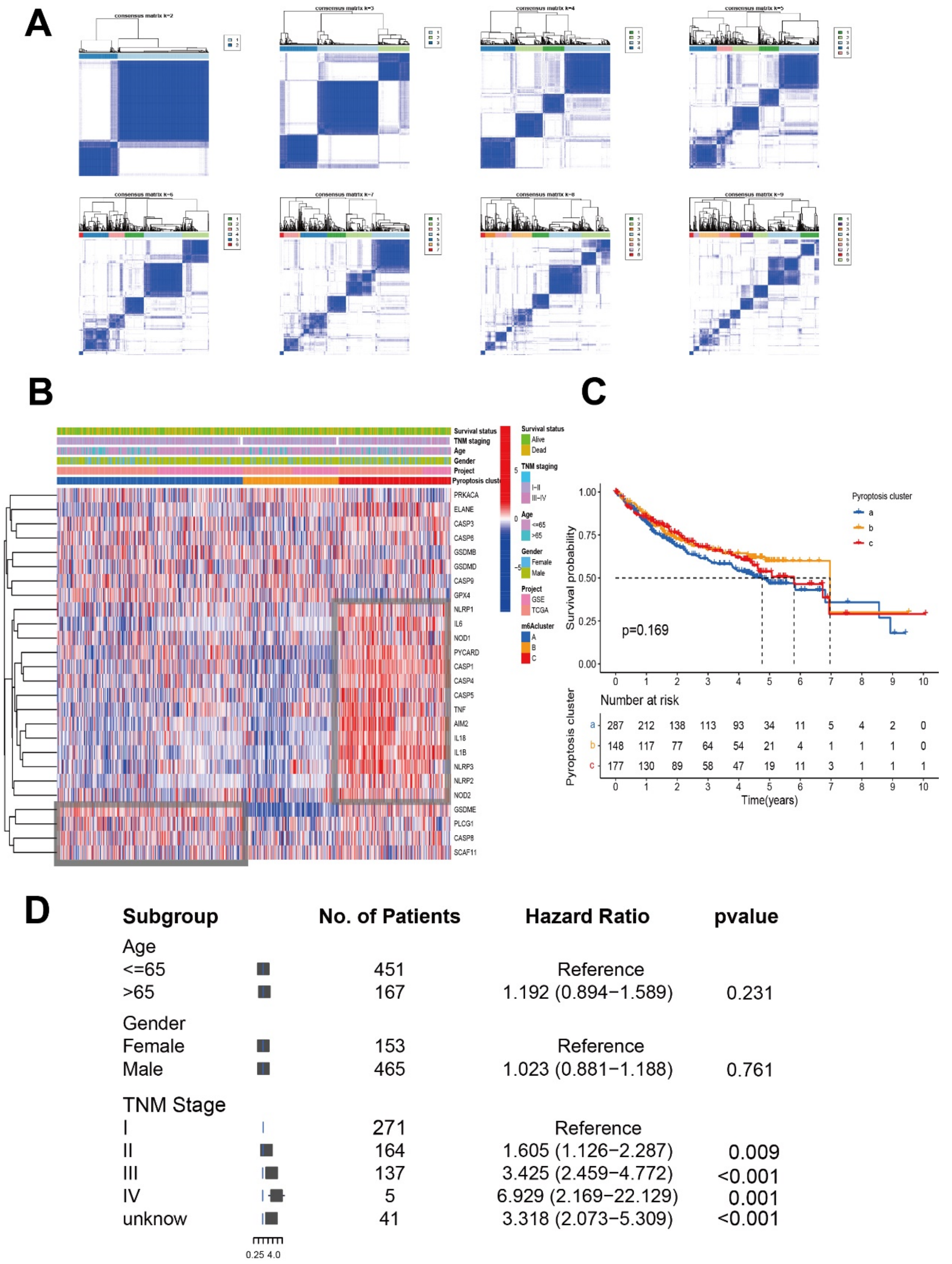
A
B
Survival status
TNM staging
Age
Gender
Project
PRKACA
ELANE
CASP3
CASP6
GSDMB
GSDMD
CASP9
GPX4
NLRP1
IL6
NOD1
PYCARD
CASP1
CASP4
CASP5
TNF
AIM2
IL18
IL1B
NLRP3
NLRP2
NOD2
GSDME
PLCG1
CASP8
SCAF11
Alive
Dead
I–II
III–IV
<=65
>65
Female
Male
GSE
TCGA
m6Acluster
C
Pyroptosis cluster
p=0.169
Survival probability
Number at risk
Time(years)
D
Subgroup
No. of Patients
Hazard Ratio
pvalue
Age
<=65 451 Reference
>65 167 1.192 (0.894−1.589) 0.231
Gender
Female 153 Reference
Male 465 1.023 (0.881−1.188) 0.761
TNM Stage
I 271 Reference
II 164 1.605 (1.126−2.287) 0.009
III 137 3.425 (2.459−4.772) <0.001
IV 5 6.929 (2.169−22.129) 0.001
unknow 41 3.318 (2.073−5.309) <0.001
0.25 4.0

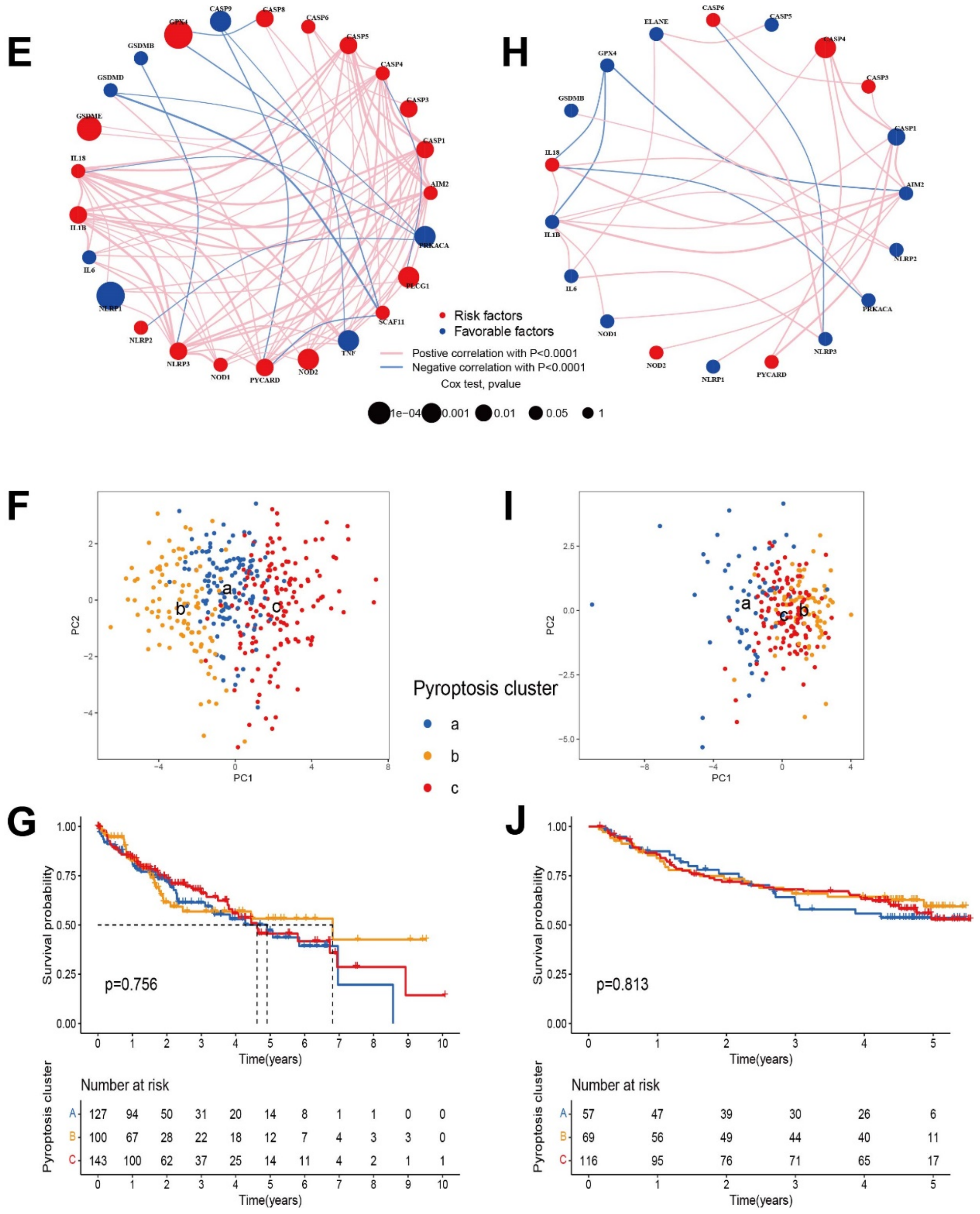

Figure S2. Characteristics of pyroptosis patterns. (**A**) the consensus distributions for k (2 to 9) based on pyroptosis genes. (**B**) Unsupervised clustering of pyroptosis genes. The clusters of clinical characteristics and pyroptosis patterns were used as sample annotations (red represents high expression level; blue, low level). (**C**) Overall survival analysis of pyroptosis clusters a (blue), b (yellow) and c (red). (**D**) Subgroup analysis estimating clinical prognostic value of patients' age, gender, and cancer stage by univariate Cox regression. The length of the horizontal line represents the 95% confidence interval for each group. The vertical dotted line represents the hazard ratio (HR) of all patients. Analyses of individual dataset (**E–G**) TCGA-LIHC, (H-J) GSE14520; (**E,H**) The correlation network of the pyroptosis-related genes (red line: positive correlation; blue line: negative correlation. The size (*p* value from 0.001 to 1) of the dot reflects the strength of the relationship between each gene and prognosis). The colors of dots represent protective (blue) and risk roles (red); Identification of potential immune subtypes of HCC based on pyroptosis genes. (**F,I**) PCA plots of clusters a (blue), b (yellow) and c (red); (**G,J**) Overall survival analysis of pyroptosis clusters a (blue), b (yellow) and c (red).

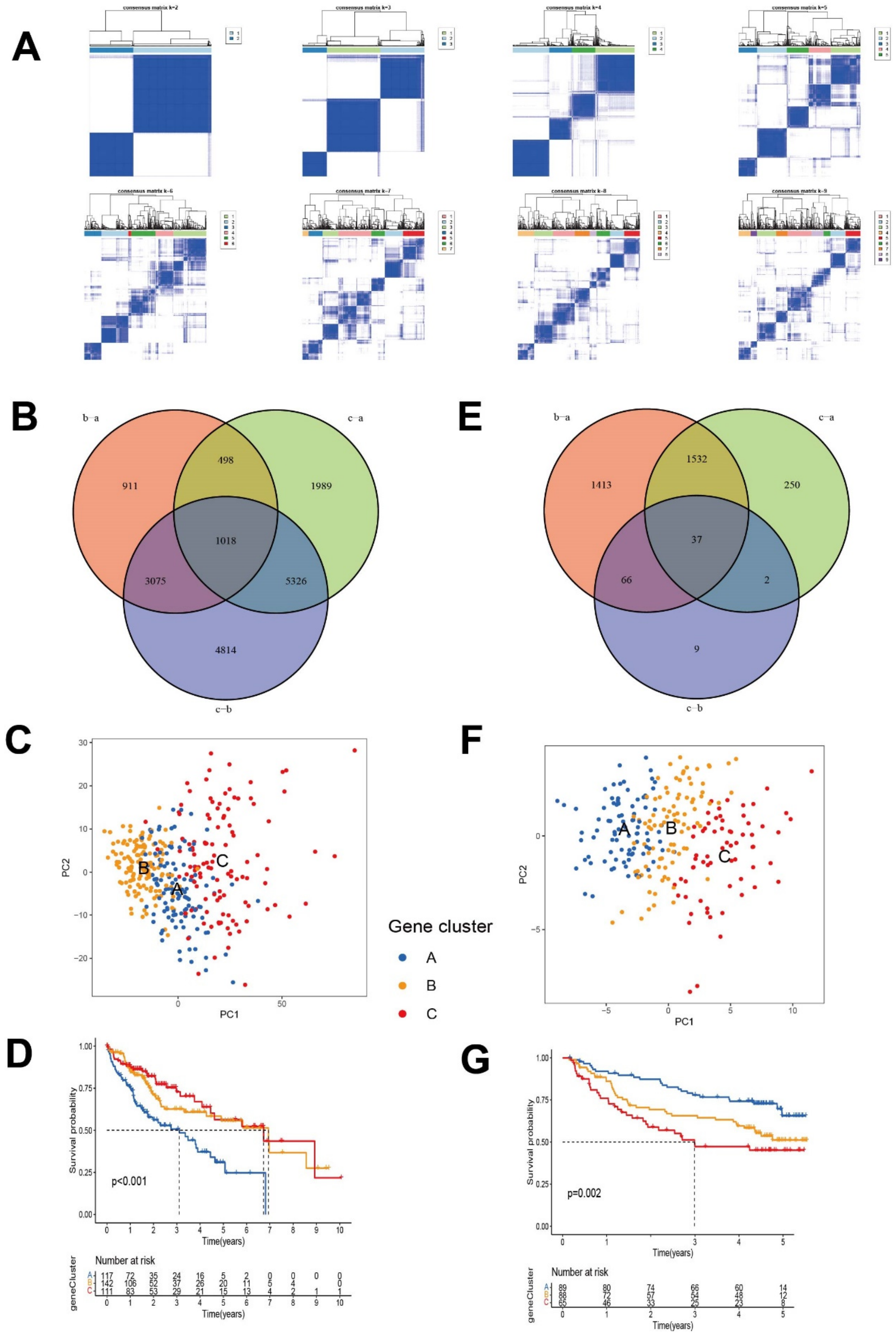

Figure S3. Identification of DEG-related subtypes. (**A**) The consensus distributions for k (2 to 9) based on screened DEGs. Separated analyses of (**B–D**) TCGA-LIHC and (**E–G**) GSE14520; (B)(E) Overlapping analyses of significant genes using a Venn diagram. (**C,F**) PCA plots of gene clusters A (blue), B (yellow) and C (red); (**D,G**) Overall survival analysis of gene clusters A (blue), B (yellow) and C (red).

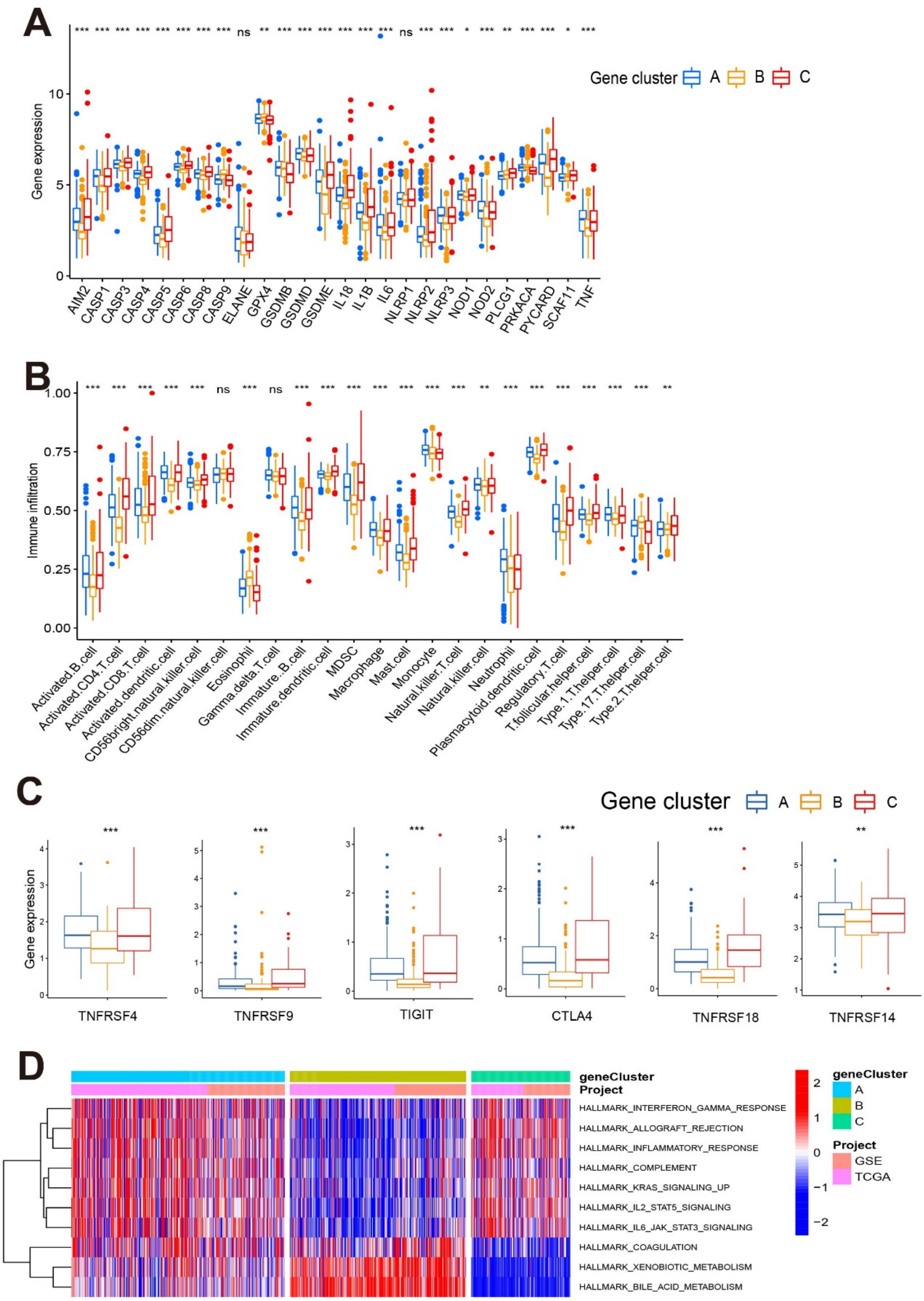

Figure S4. The expression distribution and GSVA enrichment features of gene clusters A–C. (**A**) the distribution of pyroptosis genes and (**B**) difference in the abundance of infiltrating immune cells among the three clusters. (**C**) Statistical analysis of selected immune modulators. (**D**) Heatmap distribution of the hallmark gene sets among the three clusters (red: activated pathways, blue: inhibited pathways, adjusted p value < 0.05). * $p < 0.05$, ** $p < 0.01$, *** $p < 0.001$.

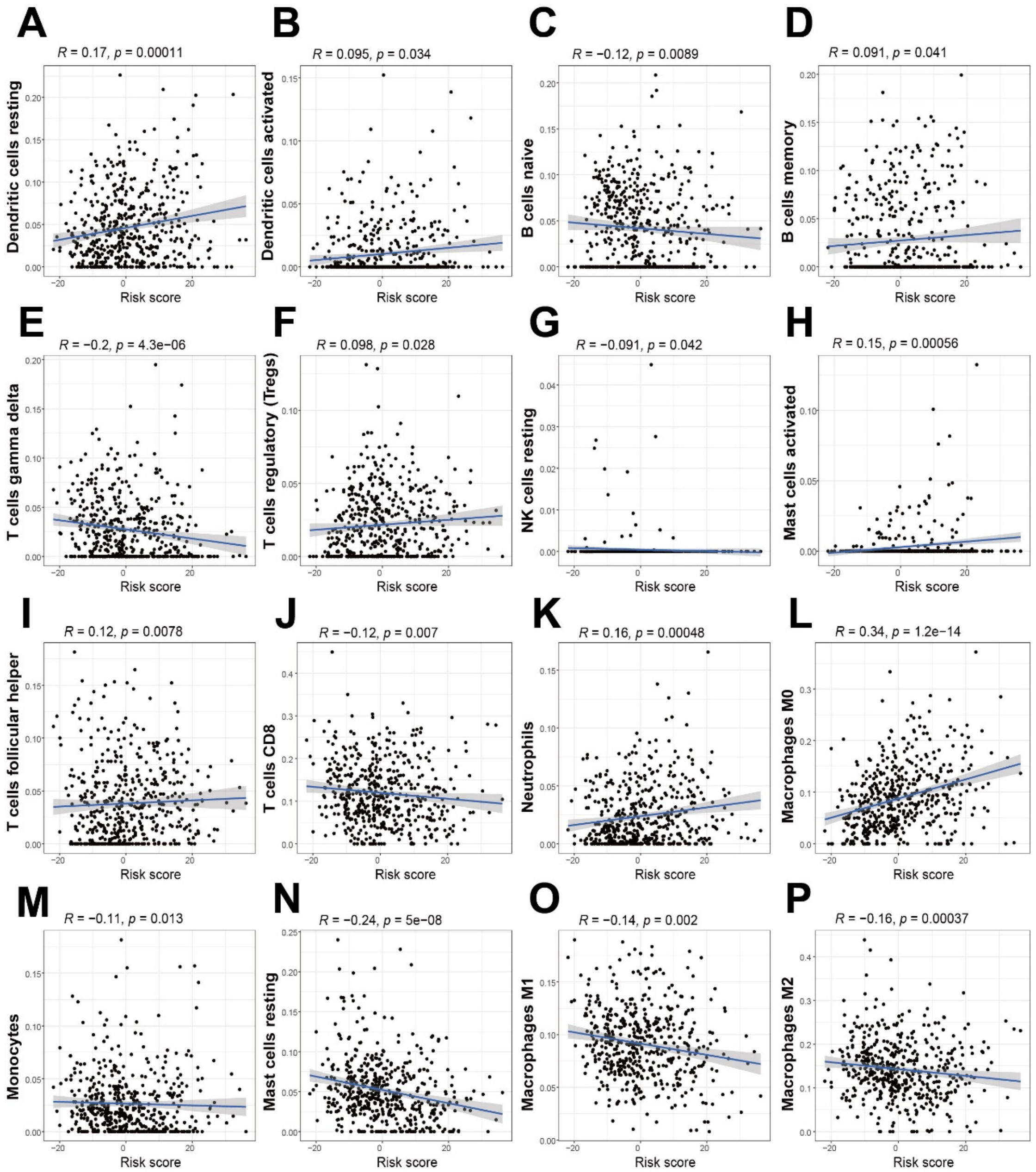

Figure S5. The association between pyroptosis risk scores and different immune subtypes (**A**) resting dendritic cell, (**B**) activated dendritic cell, (**C**) naïve B cell, (**D**) memory B cell, (**E**) gamma delta T cell, (**F**) Treg, (**G**) resting NK cell, (**H**) activated mast cell, (**I**) follicular helper T cell, (**J**) CD8+ T cell, (**K**) neutrophil, (**L**) M0 macrophage, (**M**) monocyte, (**N**) resting mast cell, (**O**) M1 macrophage, (**P**) M2 macrophage.

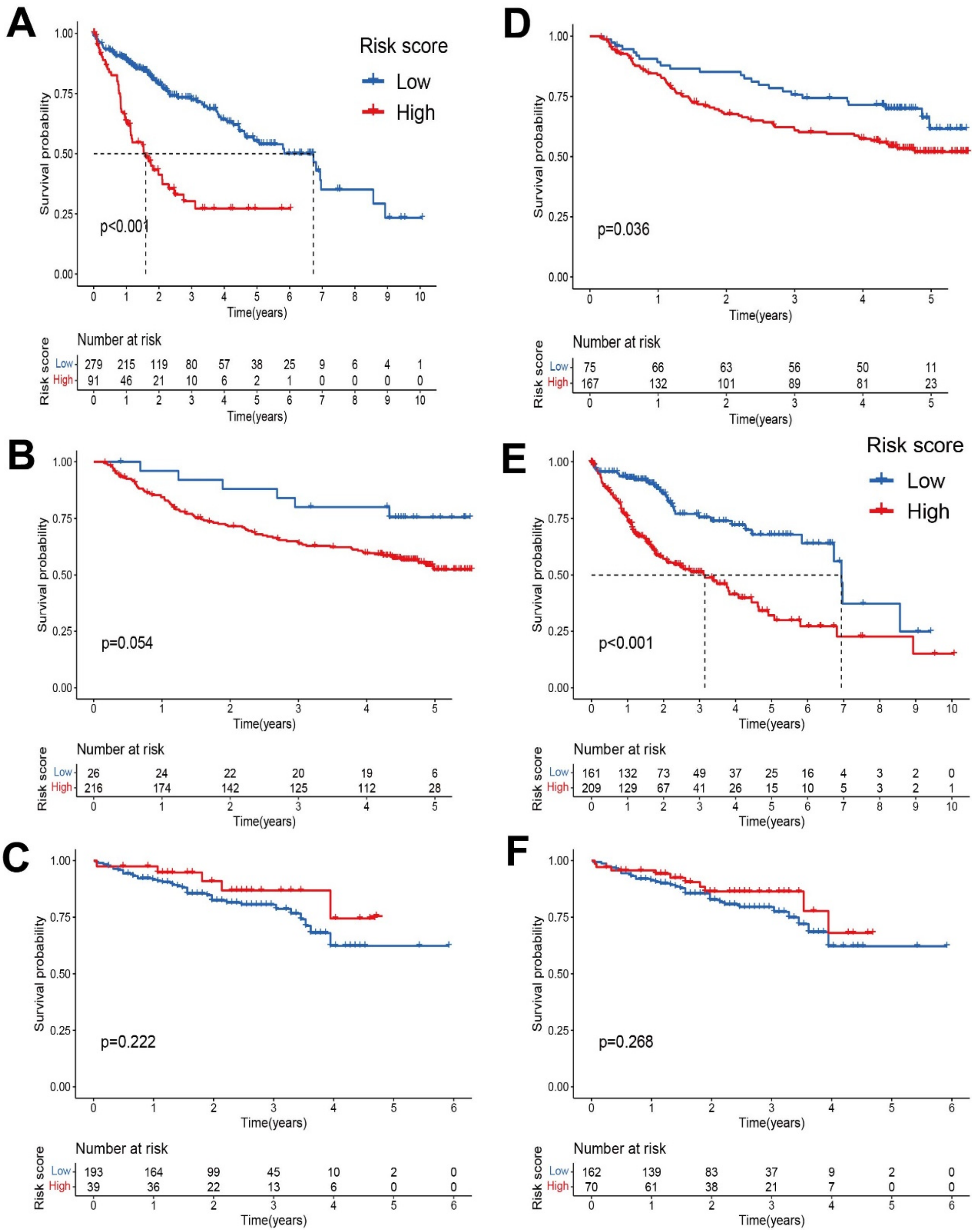

Figure S6. Cross verification of high (red)- and low (blue)- pyroptosis risk scoring system in overall survival analyses among separated databases. (**A**) Independent risk scoring classification within TCGA-LIHC, (**D**) independent risk scoring classification within GSE14520; (**B**) the efficiency of risk scoring system generated from TCGA-LIHC in classifying GSE14520 patients; (**E**) the efficiency of risk scoring system generated from GSE14520 in classifying TCGA-LIHC patients; And the further verification of the risk classification generated from TCGA-LIHC (**C**) and GSE14520 (**F**) in external ICGC data sets.

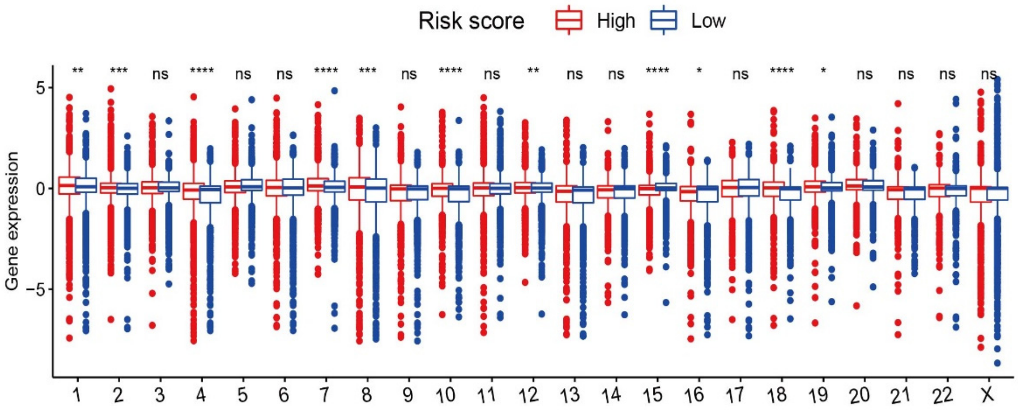

Figure S7. Statistical analysis of CNVs in whole chromosomes. * $p < 0.05$, ** $p < 0.01$, *** $p < 0.001$.
